# Global Molecular Diversity of the Halotolerant Fungus *Hortaea werneckii*

**DOI:** 10.3390/life8030031

**Published:** 2018-07-23

**Authors:** Alessia Marchetta, Bert Gerrits van den Ende, Abdullah M. S. Al-Hatmi, Ferry Hagen, Polona Zalar, Montarop Sudhadham, Nina Gunde-Cimerman, Clara Urzì, Sybren de Hoog, Filomena De Leo

**Affiliations:** 1 Department of Chemical, Biological, Pharmaceutical and Environmental Sciences, University of Messina, 98122 Messina, Italy; alessiamarchetta20@gmail.com (A.M.); urzicl@unime.it (C.U.); 2 Westerdijk Fungal Biodiversity Institute, 3584 CT Utrecht, The Netherlands; b.gerritsvandenende@westerdijkinstitute.nl (B.G.v.d.E.); abdullaalhatmi@gmail.com (A.M.S.A.-H.); f.hagen@westerdijkinstitute.nl (F.H.); s.hoog@westerdijkinstitute.nl (S.d.H.); 3 Centre of Expertise in Mycology of RadboudUMC/Canisius Wilhelmina Hospital, 6525 GA Nijmegen, The Netherlands; 4 Ministry of Health, Directorate General of Health Services, 133 Ibri, Oman; 5 Department of Biology, Biotechnical Faculty, University of Ljubljana, SI-1000 Ljubljana, Slovenia; Polona.Zalar@bf.uni-lj.si (P.Z.); nina.gunde-cimerman@bf.uni-lj.si (N.G.-C.); 6 Department of Biology, Faculty of Science and Technology, Suan Sunandha Rajabhat University, 10300 Bangkok, Thailand; sudhadham@yahoo.com

**Keywords:** *Hortaea werneckii*, epidemiology, AFLP, multilocus sequencing, haplotype networks

## Abstract

A global set of clinical and environmental strains of the halotolerant black yeast-like fungus *Hortaea werneckii* are analyzed by multilocus sequencing and AFLP, and physiological parameters are determined. Partial translation elongation factor 1-α proves to be suitable for typing because of the presence/absence of introns and also the presence of several SNPs. Local clonal expansion could be established by a combination of molecular methods, while the population from the Mediterranean Sea water also responds differently to combined temperature and salt stress. The species comprises molecular populations, which in part also differ physiologically allowing further diversification, but clinical strains did not deviate significantly from their environmental counterparts.

## 1. Introduction

Knowledge on fungi living under extreme conditions has increased significantly over the last two decades. Where general hypotheses of survival under hostile conditions concerned dormancy or refractive sporulation stages in the past, we now know of the existence of a wide array of phylogenetic diverse, highly adapted fungi that produce assimilative, growing thallic in the extreme. Examples are found in the cold [1], at high temperatures [2], with toxic hydrocarbons [3] or ultra-low pH [4], at high osmolarity [5], or on rock [6,7]. The halophilic black yeast *H. werneckii* is one of the most salt tolerant eukaryotic organisms so far described [8]. It is characterized by melanin production, pleomorphism of yeast and filamentous phases, and meristematic development [9,10]—characters we also observe in numerous rock-inhabiting fungi [6]. In culture it reproduces clonally, a sexual state is not known. The fungus has a global distribution in seawater and adjacent habitats, such as sea sponges, marine and salted freshwater fish, corals, microbial mats in salterns, beach soil, salt marsh plants and salted food [11,12,13]. The fungus is consistently encountered in deep Mediterranean Seawater [14]. The primary ecological niche of *H. werneckii* involves hypersaline waters, the fungus enriched to be dominant in solar salterns [15,16]. *Hortaea werneckii* has a broad range of growth from zero to 30% NaCl (*w*/*v*) [17,18], but with an optimum value of around 15% of salt [19]. The fungus is in use as a model for studying the molecular and physiological basis of salt tolerance in eukaryotes [17,19,20].

*Hortaea werneckii* has also been described as the cause of human tinea nigra, a superficial infection limited to the dead surface of the skin (stratum corneum), which mostly occurs in warmer climates [21,22]. The infection is mild, but patients tend to be worried because of similarity with serious skin diseases, and therefore the disorder is noteworthy. Questions are whether clinical strains differ from their environmental counterparts, and whether subtypes exist, which might allow pathogenic adaptation. For this reason, we compared growth responses at 25 and 37 °C. The present study was carried out to determine the molecular epidemiology of *H. werneckii* isolated from different sources (environmental and clinical) on a global scale, including the Mediterranean Seawater strains that were collected at a depth of up to 3402 m during two oceanographic cruises in December 2013 and March 2017. We applied several molecular typing methods, and analyzed physiological parameters.

## 2. Materials and Methods

### 2.1. Fungal Strains

Sixty-seven strains of *Hortaea werneckii* originating from a wide diversity of geographic localities and sources were considered in this study (Table 1). Twenty-five were collected from the Mediterranean Sea during the oceanographic cruises, DEEP-PRESSURE and VENUS-4, on board of the Research Vessels R/V URANIA (December 2013) and MINERVA 1 (March 2017), respectively, and maintained in the collection of the Department of Chemical, Biological Pharmaceutical and Environmental Sciences of University of Messina (Italy). Seawater was sampled from different stations located in the central and south–east of the Mediterranean Sea from the surface up to 3402 m of depth. Aliquots of sampled seawater were immediately filtered with nitrocellulose filters of a 0.45-μ pore size (Millipore, MI, Italy); filters were then placed face up on the seawater medium (1% glucose, 0.3% yeast extract, 0.3% malt extract, 0.5% peptone and 2% agar in filtered seawater) [23] and incubated at room temperature up to two weeks. After growth, enumeration of fungi was carried out as propagules/L of samples and colonies were randomly chosen and isolated on malt extract agar (MEA, Oxoid, Basingstoke, England) at 25 °C and stored on potato dextrose agar (PDA) and MEA (Oxoid) at 4 °C. Genetic and physiological comparisons were done with strains acquired from the reference collection of Centraalbureau voor Schimmelcultures (housed at the Westerdijk Fungal Biodiversity Centre, Utrecht, The Netherlands).

### 2.2. Growth at Different Salinities at 25 °C and 37 °C

Forty-four strains representative of the entire set (14 from the Mediterranean water and 30 from the CBS collection) were used for testing growth at different concentrations of salt and at 25 and 37 °C. A loopfull of colonies grown on MEA was suspended in 500 µL of sterile demineralized water and vortexed for 10–20 s at the maximum speed. The suspension was line-streaked on plates of MEA without NaCl, and with 15%, 20%, and 25% (*w*/*v*) NaCl. Colony diameters were measured after 15 days of incubation.

### 2.3. DNA Extraction and Sequencing

Genomic DNA was extracted following the cetyltrimethylammonium bromide (CTAB) protocol [24]. DNA was quantified with a NanoDrop^®^ ND-1000 Spectrophotometer (Thermo Fisher, Wilmington, NC, USA), and samples were stored at −20 °C until use. Internal transcribed spacer region (ITS) and the partial translation elongation factor-1α (*TEF1*) were amplified with primer pairs—ITS1 and ITS4 [25] and EF1-728F [26] and EF1-1567R [27], respectively. PCR mixtures were prepared as previously described [28]. Cycling conditions included 95 °C for 5 min, followed by 30 cycles consisting of 95 °C for 45 s, 48 °C for 30 s and 72 °C for 1 min, and a post-elongation step at 72 °C for 8 min for ITS; and one cycle of 5 min at 95 °C, followed by 40 cycles of 45 s at 94 °C, 35 s at 52 °C and 1.20 min at 72 °C and a post-elongation step of 8 min at 72 °C for *TEF1*. PCR products were visualized by electrophoresis on 1% (*w*/*v*) agarose gels. PCR amplicons were sequenced in both directions using standard conditions with a BigDye^TM^ v3.1 terminator cycle sequencing kit (Applied Biosystems, Bleiswijk, The Netherlands). Sequences were edited using SeqMan in the Lasergene package (DNAstar, Madison, WI, USA). GenBank accession numbers are shown in Table 1.

### 2.4. Amplified Fragment Length Polymorphism Genotyping

Amplified Fragment Length Polymorphism (AFLP) genotyping was done as previously described [29]. In brief, genomic DNA was subjected to restriction-ligation with a mixture containing a HpyCH4 IV adapter, a MseI adapter, 2U of HpyCH4 IV, 2U of MseI and 1U of T4 DNA ligase for 1 h at 20 °C. Reaction products were diluted and combined with a size marker, Orange600 (Nimagen, The Netherlands), followed by a heating step for 1 min at 100 °C and cooling down to 4 °C. Fragment analysis was carried out using an ABI3500xL Genetic Analyzer (Applied Biosystems). Data were evaluated using BioNumerics v7.5 (Applied Maths, Sint-Martens-Latem, Belgium) via UPGMA clustering with the Pearson’s correlation coefficient. Only DNA fragments in the range of 40–400 bp were taken into account.

### 2.5. Haplotype Networks

The distribution of haplotypes based on sequences data of *TEF1* of the studied isolates was determined by PopArt v1.7 [30]. Briefly, a haplotype nexus-file was created with DnaSP 5.10 [31]. Gaps in the alignment were not considered creating haplotypes based on nucleotide differences. The file was manually prepared for PopArt to add the geographical origin of the isolates, if known. A median joining network was created. The lengths of the lines in the network do not correspond with the nucleotide differences, which are indicated by tickmarks and connectors.

## 3. Results

### 3.1. Sequencing

Sequencing of the rDNA ITS region and subsequent comparison in GenBank and in an in-house black yeast database maintained at the Westerdijk Institute confirmed all 67 strains belong to *Hortaea werneckii*, with identity ≥ 99%, e-value: 0, without gaps. Sixteen SNPs were detected in the dataset. Sequences of the partial *TEF1* gene were aligned with MAFFT version 7.402 (https://mafft.cbrc.jp) followed by manual adjustment with MEGA v.7. The resulting alignment revealed the presence of one, two or none introns in the partial sequence. The strains from the Mediterranean Sea maintained Intron 1, while Intron 2 was missing, as in the environmental strains CBS 117931 from Spain and CBS 100456 from Slovenia. The tinea nigra isolates from Mexico, the type strain CBS 107.67 and seven other strains from different geographical areas and sources (CBS 373.92, CBS 116.30, CBS 117.90, CBS 706.76, CBS 705.76, CBS 359.66, 708.76 and CBS 120952) maintained both introns (Intron 1 and Intron 2). No introns were detected in the tinea nigra isolates CBS 111.31 from Brazil and CBS 100455, CBS 116.90, CBS 115.90, CBS 123850, CBS 132932, CBS 132931, CBS 132911, CBS 132930, CBS 110352, CBS 100496, CBS 100457 and CBS 255.96 from other environmental sources. The strains from deep sea showed a relatively large number of double peaks in the electrophorograms; these occur at regular places in the alignment in 19 positions. The minimum of 2 double peaks in strain MC 857 and the maximum of 16 double peaks in strain MC 870 were observed. All stains from the Mediterranean Sea had double peaks in 2 of these positions, which are probably the result of the diploid and highly heterozygous genome observed in many *H. werneckii* strains [32]. In the present study, the highest peak was chosen for the haplotype network. The strains contain Intron 1 of 55 bp long, except for CBS 373.92, CBS 116.30, CBS 117.90, CBS 705.76, CBS 706.76, CBS 708.76 and CBS 359.66, which are 53 bp long. Intron 2 shows more diversity in length. CBS 120952 from Puerto Rico has a length of 90 bp. Other strains contain a shorter Intron 2: CBS 373.92 is 66 bp long; all CBS 116.30, CBS 117.90, CBS 705.76, CBS 706.76, CBS 708.76 and CBS 359.66 are 69 bp, while CBS 107.67, CBS 122340, CBS 123041, CBS 123045, CBS 122344, CBS 123043, CBS 123044, CBS 123046, CBS 126986, CBS 126984, CBS 126985 and CBS 123042 have a length of 63 bp.

Based on the number of SNPs in the introns, strains could be assigned to three main groups. One group is represented by the strains from the Mediterranean Sea, which differ from each other by 0‒4 SNPs in the sequences of their unique intron (Intron 1). A second group is formed by the tinea nigra isolates from Mexico and the type strain CBS 107.67, of which Intron 1 and Intron 2 sequences had no SNPs, except for CBS 123042, which showed one SNP of difference in Intron 2 and CBS 107.67 with one SNP in Intron 1. The last group is represented by CBS 116.30, CBS 117.90, CBS 706.76, CBS 705.76, CBS 359.66 and CBS 708.76 of the environmental and clinic origin, of which sequences of Intron 1 and Intron 2 had no SNP.

Some strains could not be assigned to any of the three main groups: isolates CBS 100456 from Slovenia and CBS 117931 from Spain had only Intron 1, similar to strains from the Mediterranean Sea, but they differed from these by 9–14 SNPs. Strain CBS 373.92 from Spain had two introns, as well as the strains from Mexico and the strains of the third group, but differed in 4 and 6 SNPs in Intron 1 and in 21‒22 and 9 SNPs in Intron 2, respectively. The environmental isolate CBS 120952 from Puerto Rico showed a large range of diversity compared with the other strains: from 5 to 14 SNPs of difference in Intron 1 and from 48 to 52 SNPs in Intron 2. For sequences CBS 122.32, CBS 410.51, CBS 707.76, CBS 126.35, CBS 122342, CBS 126987, and CBS 122348, it was not possible to verify the presence of introns because they were too short.

### 3.2. Physiology

Growth responses at different salt concentrations and at two temperatures are summarized in Table 2. At 25 °C, all strains considered were able to grow on MEA without addition of NaCl. The majority of strains grew up to a NaCl concentration of 25% in the medium, except for CBS 107.67, CBS 116.30, CBS 126.35, CBS 123041, CBS 110352 and CBS 410.51 where limited growth (<4 mm) was observed at 20% NaCl. Excellent growth (≥10 mm/15 d) occurred in all isolates from the Mediterranean Sea on the medium without additional NaCl, with 15% NaCl colony expansion dropped to 4–6 mm/15 d. The majority of clinical strains and strains from environmental habitats grew equally well at 0% and 15% of additional NaCl, with no or little difference between these salt concentrations: 15/30 strains were indifferent on these conditions, 9/30 strains were inhibited, and 6/30 were stimulated at 15% NaCl.

At 37 °C, the majority of all strains investigated was able to grow in the range from 0% to 20% NaCl, while none grew in NaCl with a concentration of 25% (*w*/*v*) (Table 2). Mediterranean Sea strains grew much slower (4–6 mm/15 d) than at 25 °C, but all except one grew equally well with 0% and 15% of additional NaCl. None grew at 37 °C at the concentration of 25% NaCl. Remaining clinical and environmental strains showed poor or no growth on media without additional NaCl, but invariably grew significantly better with 15% salt: in six strains, the stimulus was from zero to ≥ 10 mm diam/15 d. Thirteen strains, among which three clinical strains, i.e., CBS 116.30, CBS 708.76 and CBS 126.35, grew at 37 °C exclusively when incubated on media with 15% or 20% of additional NaCl.

### 3.3. Amplified Fragment Length Polymorphism Genotyping

**Figure 1:**
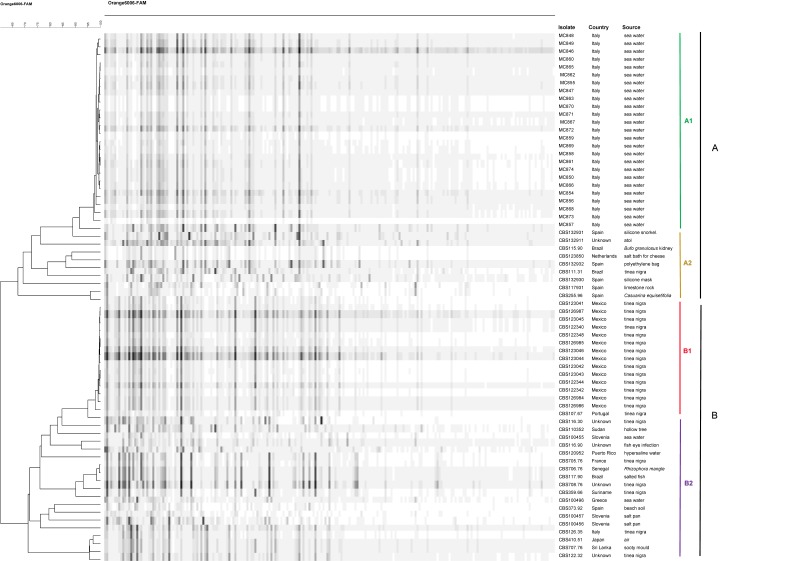
Clustering of the amplified length polymorphism banding pattern of the 67 *Hortaea werneckii* isolates. The dendrogram is generated using the Unweighted Pair Group Method with Arithmetic Mean algorithm.

Frequently cutting restriction endonucleases HpyCH4IV and MseI and two selective primers were used in the AFLP analysis to fingerprint the genomes of 67 strains of *H. werneckii* (Figure 1). Two main clusters, A and B, were apparent at a profile similarity of 60%. Several subgroups were observed within the two main clusters. All isolates from the Mediterranean Sea grouped in cluster A (subgroup A1), representing the largest part of the strains in that cluster (25/34). Nearly all Mediterranean strains were very close, with similarities ranging from 97% to 100%, but strain MC 857 had a similarity of 90% to its nearest neighbor in group A. Cluster A also comprised seven strains of the environmental origin from Europe, Brazil, and from an unknown origin, plus a tinea nigra strain from Brazil CBS 111.31 (subgroup A2).

Cluster B included all *H. werneckii* isolates from tinea nigra in Mexico, which were highly similar to each other (97–100%). These strains showed high genetic relatedness (~95%) to the *H. werneckii* type strain, CBS 107.67 from tinea nigra in Portugal (subgroup B1). Cluster B further comprised 12 environmental strains from Brazil, Greece, Japan, Puerto Rico, Senegal, Slovenia, Spain, Sri Lanka, Sudan, and one from an unknown country, in addition to six clinical strains from France, Italy, Suriname and from unknown countries (subgroup B2).

### 3.4. Haplotype Network

**Figure 2:**
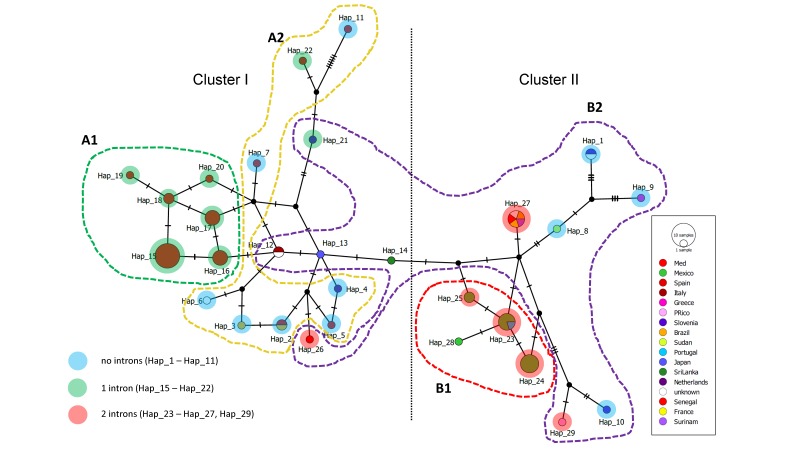
Haplotype networking generated on the basis of the SNPs in *TEF1* sequence data. Presence/absence of introns and AFLP groups is shown.

On the basis of SNPs in *TEF1* sequence data, 29 haplotypes could be distinguished. These were plotted in a network, where presence/absence of two introns is shown (Figure 2). Two main clusters of closely interrelated haplotypes could be distinguished, I and II, with Hap 11 at a considerable distance, and Haps 1, 9, 10 and 29 loosely affiliated to cluster II. Members of both clusters were geographically diverse, but strains from the Mediterranean Sea in cluster I and from Mexico in cluster II were closely linked with the maximum of one to two mutations between strains. Haplotype 27 showed a high rate of geographical diversity, with isolates from Senegal, Brazil, Surinam, France and an unknown country.

Results obtained from sequencing of partial *TEF1* and from AFLP are almost concordant. The two main clusters, A and B, obtained from the AFLP analysis matched, with some exceptions, with the two clusters observed in the Haplotype network of *TEF1* (Figure 2). In fact, the strains presented in AFLP cluster A grouped in different haplotypes of cluster I in the network, except for CBS 100456 (Hap 21), CBS 126.35 and CBS 122.32 (Hap 12), CBS 410.51 (Hap 13), CBS 707.76 (Hap 14) and CBS 373.92 (Hap 26), which were in cluster B in the AFLP dendrogram. The remaining strains comprised in AFLP cluster B matched perfectly with cluster II of the network. The presence/absence of the introns in the partial *TEF1* sequence followed the distribution of the strains in the two clusters of AFLP and the network: the results showed that the strains which had only Intron 1 grouped in AFLP cluster A and the network cluster I, while strains with two introns were present in AFLP clusters B and network cluster II. Two exceptions are CBS 100456 (Hap 21) from Slovenia with one intron that was in cluster B of the AFLP dendrogram and CBS 373.92 (Hap 26) with 2 introns that was in cluster I of the network. The strains without introns in the partial sequence of *TEF1* had variable distribution: the strains from Spain CBS 132931 (Hap 2), CBS 132932 (Hap 5), CBS 132930 (Hap 7), CBS 255.96 (Hap 11), one from an unknown country CBS 132911 (Hap 6), two from Brazil CBS 115.90 (Hap 3) and CBS 111.31 (Hap 2) and CBS 123850 from The Netherlands (Hap 4) were present in AFLP cluster A and network cluster I, while two strains from Slovenia CBS 100455 (Hap 1) and CBS 100457 (Hap 10), CBS 110352 (Hap 8) from Sudan, CBS 100496 (Hap 9) from Greece and one from an unknown country CBS 116.90 (Hap 1) were in AFLP cluster B and in network cluster II.

## 4. Discussion

*Hortaea werneckii* is a halophilic black yeast in the order Capnodiales which is phylogenetically clearly demarcated from its nearest neighbor, *Penidiella venezuelensis* [33]. Its sexual cycle is still unknown. Whole genome analyses of *H. werneckii* showed the presence of the mating type idiomorph MAT1-1, while MAT1-2 was absent, suggesting that the species is heterotallic [32,34]. The fungus has an exceptionally large, duplicated genome [32] which interferes with sequencing of most housekeeping genes because of amplification of mutated alleles in a single strain. Among the available epidemiological typing techniques, AFLP stands out as an option for the analysis of genetic diversity between populations. Sequence data of partial *TEF1* proved to be significant because of the occurrence of numerous SNPs, and of the absence or presence of two introns, while ITS rDNA contains only a limited number of SNPs. These data are summarized in Figure 2. Combining the applied markers, significant intra-specific diversity was revealed, which may shed light on the species’ routes of dissemination.

*Hortaea werneckii* has a global distribution in haline habitats. The fungus occurs at low density in seawater [35], but emerges exponentially in (sub)tropical coastal areas when salt levels increase until the saturation point is reached [19]. The fungus is extremely abundant in natural and artificial crystallization pans. Another ecological niche of the fungus has been published as human skin. The fungus causes black spots on the palms of human hands, known as ‘tinea nigra’, and for this reason, *Hortaea* has been considered as a human pathogen in the past [36]. However, Göttlich et al. [37] noted that the disorder by this non-keratinolytic fungus is nearly confined to hands and sometimes feet of hyperhydrotic individuals after a day at the beach, and thus asymptomatic colonization of exceptionally salty skin was a likely explanation of this phenomenon. *Hortaea werneckii* thus can be viewed as a fungus unambiguously associated with high salt. Infraspecific diversification might allow adaptation to the habitat of the human skin. In the present study, we observed that most strains, when subjected to temperature stress of 37 °C, grew much better when they were subjected to salt stress of 15% of NaCl, underlining its halophilic nature; apparently, the fungus does not experience the increased salt level as stress, but rather as its optimal condition. This was, however, not the case with the strains from the Mediterranean Sea. A possible explanation is that most strains were derived from deep seawater, which may be less often related to the hypersaline environment of coastal saltpans. Clinical strains were not located in this clade, and did not deviate significantly from their environmental counterparts; no adaptive trend to the human host was revealed.

Global distribution of *H. werneckii* was studied by combined partial *TEF1* sequencing and AFLP profiling. *TEF1* proved to be highly suitable to characterize populations, not only by a number of SNPs, but also by two introns that were independently absent or present. AFLP patterns showed intraspecific diversity with two main groups of profiles. This level of diversity is higher than that known in, for example, *Cryptococcus*, where profiles are nearly monomorphic within sibling species [38], or in anthropophilic dermatophytes [39]. High intraspecific diversity is known, for example, in *Aspergillus fumigatus*, where strains, even when sampled on a single location, may deviate significantly from each other [40]. In our AFLP dataset, several groups of strains with nearly identical profiles clones and limited geographic distributions were recognizable. Particularly, the strains from the Mediterranean Sea proved to be highly similar. This was corroborated by their response to salt stress, which deviated from that of the remaining strains. In addition, some isolates from Mexico, all from human tinea nigra were highly similar. Intron distribution profiles reinforce similarity of geographically adjacent isolates. This level of molecular similarity and close geographic vicinity of isolates suggests local, possibly clonal dispersal. An important exception is Haplotype 27, containing strains from Europe, Africa and South America.

The group of strains from the Mediterranean Sea consistently deviates from remaining strains, not only molecularly but also by being insensitive to salt stimulation for survival at temperature stress. This group is thus less clearly halophilic. The AFLP dendrogram showed that there is no genetic variability among the strains originating from different stations and depths. This ecologically relevant difference might allow separating the evolution of this cluster of strains, which, however, does not imply any clinical adaptation.

## Figures and Tables

**Table 1:** Geographic origin, source of isolation, ITS and *TEF1* accession numbers of *Hortaea werneckii* strains included in this study.

Collection Number	Country	Source	Gen Bank Accession Number	
			ITS	TEF1 *
CBS 100455	Slovenia	Seawater	AY128704	MH259543
CBS 100456	Slovenia	Salt pan, saline water	MH028914	MH259581
CBS 100457	Slovenia	Salt pan, saline water	MH028913	MH259579
CBS 100496	Greece	Sea-sprayed marble	AY128703	MH259542
CBS 107.67 T	Portugal	Tinea nigra	AJ238468	MH259537
CBS 110352	Sudan	Hollow tree	MH028917	MH259577
CBS 111.31	Brazil	Tinea nigra	AJ238679	MH259546
CBS 115.90	Brazil	*Bufo granulosus* kidney	AJ238470	MH259548
CBS 117.90	Brazil	*Osteoglossum bicirrhosum*	AJ238472	MH259526
CBS 116.30	Unknown	Tinea nigra	MH028923	MH259521
CBS 116.90	Unknown	*Chantarus chantarus* eye infection	AJ238471	MH259544
CBS 120952	Puerto Rico	Hypersaline water	MH028918	MH259519
CBS 122.32	Unknown	Tinea nigra	AJ238473	MH259574
CBS 122340	Mexico	Tinea nigra	MH028912	MH249534
CBS 122342	Mexico	Tinea nigra	MH028899	MH259529
CBS 122344	Mexico	Tinea nigra	MH028900	MH259532
CBS 122348	Mexico	Tinea nigra	MH028911	MH259528
From CBS 123041 to CBS 123046	Mexico	Tinea nigra	From MH028901 to MH028906	MH259535, MH259540, MH259538, MH259533, MH259531, MH259536
From CBS 126984 to CBS 126987	Mexico	Tinea nigra	MH028907, MH028909, MH028910, MH028908	n.d., MH259530, MH259539, MH259527
CBS 123850	Netherlands	Salt bath for salting cheeses	MH028916	MH259550
CBS 126.35	Italy	Tinea nigra	MH028921	MH259573
CBS 132911	Unknown	Atol	MH028924	MH259547
CBS 132930	Spain	Silicone scuba diving mask	MH028925	MH259578
CBS 132931	Spain	Silicone snorkel	MH028926	MH259549
CBS 132932	Spain	Polyethylene plastic bag	MH028927	MH259576
CBS 255.96	Spain	*Casuarina equisetifolia*	MH028928	MH259541
CBS 117931	Spain	Limestone rock	MH028898	MH259580
CBS 373.92	Spain	Beach soil	AJ238474	MH259520
CBS 359.66	Suriname	Tinea nigra palmaris	AJ244249	MH259524
CBS 410.51	Japan	Air	MH028919	MH259571
CBS 705.76	France	Tinea nigra	MH028920	MH259522
CBS 706.76	Senegal	*Rhizophora mangle* leaf	MH028955	MH259523
CBS 707.76	Sri Lanka	Sooty mould	MH028915	MH259572
CBS 708.76	Unknown	Tinea nigra	MH028922	MH259525
MC 846 and MC 847	Italy	Seawater (Mediterranean Sea, depth 25 m, “Vector” station)	KX427192 KX427193	MH259569 MH259545
MC 848	Italy	Seawater (Mediterranean Sea, depth 2500 m, “Vector” station)	KX427194	n.d.
MC 849	Italy	Seawater (Mediterranean Sea, depth 200 m, “KM3” station)	KX427195	MH259551
MC 850	Italy	Seawater (Mediterranean Sea, depth 94 m, “Medee” station)	KX427196	MH259582
From MC 854 to MC 859	Italy	Seawater (Mediterranean Sea, depth 0 m, “Sn2” station)	From MH028934 to MH028939	MH259558, MH259557, MH259555, MH259567, MH259554, MH259556
From MC 860 to MC 862	Italy	Seawater (Mediterranean Sea, depth 100-250 m, “Sn2” station)	From MH028940 to MH028942	MH259559, MH259570, MH259566
MC 863	Italy	Seawater (Mediterranean Sea, depth 2218 m, “Sn2” station)	MH028943	MH259561
From MC 865 to MC 874	Italy	Seawater (Mediterranean Sea, depth 3402 m, “Geostar” station)	From MH028944 to MH028953	MH259575, MH259565, MH259552, MH259560, MH259553, MH259564, MH259568, MH259563, n.d., MH259562

T = type strain. * *TEF1* accession numbers for strains CBS 126984, MC 848 and MC 873 were not determined.

**Table 2:** Growth ^1^ at different salinities at 25 °C and 37 °C.

		25 °C				37 °C		
	0% NaCl	15% NaCl	20% NaCl	25% NaCl	0% NaCl	15% NaCl	20% NaCl	25% NaCl
**Strains from the Mediterranean Sea**								
MC 846	++++	+++	+	w	++	+++	+	-
MC 848	++++	++	+	w	++	++	+	-
MC 849	++++	++	+	w	++	++	+	-
MC 850	++++	++	+	w	++	++	+	-
MC 854	++++	++	+	w	++	++	+	-
MC 858	++++	++	+	w	++	++	+	-
MC 859	++++	++	+	w	++	++	+	-
MC 860	++++	++	+	w	++	++	+	-
MC 861	++++	++	+	w	++	++	+	-
MC 862	++++	++	+	w	++	++	+	-
MC 863	++++	++	+	w	++	++	+	-
MC 865	++++	++	+	w	++	++	+	-
MC 873	++++	++	+	w	++	++	+	-
MC874	++++	++	+	w	++	++	+	-
**Clinical strains**								
CBS 107.67	++	+++	+	-	-	++++	+	-
CBS 111.31	++++	++++	++	w	+	++++	++	-
CBS 116.30	++++	+	+	-	-	+	-	-
CBS 122.32	+++	+++	++	w	+	+++	++	-
CBS 126.35	++++	++	+	-	-	+	-	-
CBS 359.66	++	++	+	w	-	++	+	-
CBS 705.76	+++	++++	+++	w	+	++++	++	-
CBS 708.76	++++	+++	++	w	-	+	-	-
CBS 122348	++++	+++	+	w	++	++++	++	-
CBS 123041	++++	++	+	-	++	++++	++	-
CBS 123046	+++	+++	+	w	++	++++	+++	-
CBS 126987	+++	++	+	w	-	++++	+	-
**Strains from salterns**								
CBS 100455	+++	++	+	w	-	++	+	-
CBS 100457	++++	++++	+	w	++	+++	+	-
CBS 120952	++++	++++	++	+	-	++++	+	-
**Strains from temperate and Mediterranean climatic zone**								
CBS 100496	+++	++	+	w	++	++++	+	-
CBS 117931	+++	++++	++	w	-	+	+	-
CBS 123850	++++	++++	++++	+	++	++++	+++	-
CBS 132931	+++	++	+	+	+	++	+	-
CBS 132932	++	++	+	w	w	+	+	-
CBS 255.96	++++	++++	++	+	-	+	+	-
CBS 373.92	++++	++++	++++	+	-	++++	+++	-
CBS 410.51	++++	++++	++	-	+	+++	++	-
CBS 706.76	+++	++++	+++	w	+	++++	++	-
**Strains from tropical and arid climatic zone**								
CBS 115.90	+++	+++	+	w	-	+	+	-
CBS116.90	+++	++++	++	w	++	++++	++	-
CBS 117.90	++++	++++	+++	w	+	++++	++	-
CBS 110352	++++	++++	+++	-	-	++++	+	-
CBS 707.76	+++	++++	+++	+	++	++++	++	-
CBS 132911	++++	++++	+++	+	-	++++	+	-

^1^ Range of growth: 1–3 mm = +; 4–6 mm = ++; 7–9 mm = +++; ≥ 10 mm = ++++; w = weak.
